# Painful rib hump: a new clinical sign for detecting intraspinal rib displacement in scoliosis due to neurofibromatosis

**DOI:** 10.1186/1748-7161-1-10

**Published:** 2006-06-14

**Authors:** Andreas Gkiokas, Socratis Hadzimichalis, Elias Vasiliadis, Marina Katsalouli, Georgios Kannas

**Affiliations:** 1 1^st ^Orthopaedic Department, Children's Hospital "P.&A. Kyriakou", Thivon & Levadias, Goudi, 11527, Athens, Greece; 2 Neurosurgery Department, Children's Hospital "P.&A. Kyriakou", Thivon & Levadias, Goudi, 11527, Athens, Greece; 3 Neurology Department, Children's Hospital "P.&A. Kyriakou", Thivon & Levadias, Goudi, 11527, Athens, Greece

## Abstract

**Background:**

Spinal cord compression and associate neurological impairment is rare in patients with scoliosis and neurofibromatosis. Common reasons are vertebral subluxation, dislocation, angulation and tumorous lesions around the spinal canal. Only twelve cases of intraspinal rib dislocation have been reported in the literature. The aim of this report is to present a case of rib penetration through neural foramen at the apex of a scoliotic curve in neurofibromatosis and to introduce a new clinical sign for its detection.

**Methods:**

A 13-year-old girl was evaluated for progressive left thoracic kyphoscoliotic curve due to a type I neurofibromatosis. Clinical examination revealed multiple large thoracic and abdominal "cafe-au-lait" spots, neurological impairment of the lower limbs and the presence of a thoracic gibbous that was painful to pressure at the level of the left eighth rib (Painful Rib Hump). CT-scan showed detachment and translocation of the cephalic end of the left eighth rib into the adjacent enlarged neural foramen. The M.R.I. examination of the spine showed neither cord abnormality nor neurogenic tumor.

**Results:**

The patient underwent resection of the intraspinal mobile eighth rib head and posterior spinal instrumentation and was neurologically fully recovered six months postoperatively.

**Conclusion:**

Spine surgeons should be aware of intraspinal rib displacement in scoliotic curves in neurofibromatosis. Painful rib hump is a valuable diagnostic tool for this rare clinical entity.

## Background

Neurofibromatosis (NF) is an autosomal dominant hereditary disorder that was first described by Frederick Daniel von Recklinghausen and is associated with skeletal, skin and soft tissue abnormalities. The most common skeletal manifestations in NF are scoliosis and kyphoscoliosis due to dystrophic osseous changes [1].

The typical spinal deformity in NF has been described as a progressive short, sharp, angular kyphoscoliotic curve [2]. Right thoracic curves predominate in scoliosis in NF but there is an increasing incidence of left thoracic curves compared to idiopathic scoliosis [3].

Rib dislocation into the spinal canal that causes spinal cord compression in patients with NF and scoliosis has previously been described [4-10]. The present report introduces a new clinical sign for detection of this rare clinical entity.

## Case report

**Figure 1:**
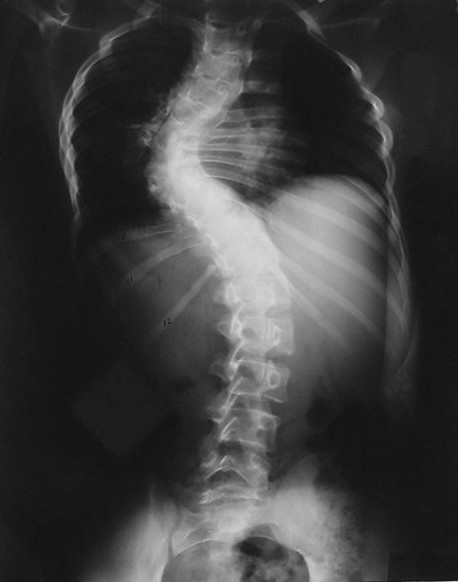
Preoperative standing posteroanterior radiograph showing left dystrophic scoliosis measuring 75 degrees from T4 to the T10.

**Figure 2:**
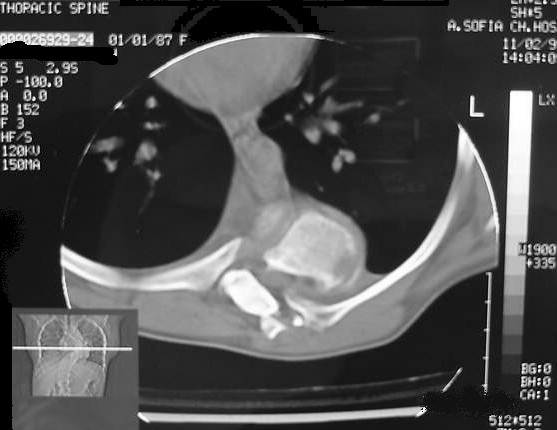
Preoperative CT scan at the level of T8 shows intraspinal dislocation of the left eighth rib head through neural foramen.

A 13-year-old girl was referred for evaluation of a progressive left thoracic kyphoscoliotic curve (Figure 1) due to a type I NF. Clinical examination revealed multiple large thoracic and abdominal "cafe-au-lait" spots and the presence of a thoracic gibbous that was painful to pressure (*Painful Rib Hump*). Neurological examination revealed difficulty in gait, bilateral Babinski and ankle clonus, right drop foot, decreased superficial sensation, and increased tendon reflections in the lower limbs. Also the child complained of daytime uncontrolled micturitions. Plain radiographs showed a single, sharp, left thoracic kyphoscoliotic curve from T4 to T10 measuring 75° by the Cobb method with the apex at eighth thoracic vertebra. CT-scan revealed detachment and translocation of the cephalic end of the left eighth rib into the adjacent enlarged neural foramen. The costovertebral joint dislocation was at the apex of the curve on the convex side and the rib head was displaced into the spinal canal and compressed the spinal cord (Figure 2). The M.R.I. examination of the spine revealed the spinal cord compression by the displaced rib head and showed no other cord abnormality or neurogenic tumor.

**Figure 3:**
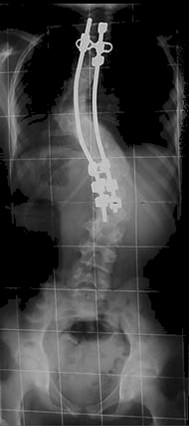
Postoperative standing posteroanterior radiograph showing the levels of instrumentation and the correction of the curve.

The patient underwent resection of the intraspinal mobile eighth rib head through a posterior approach and simultaneous correction and fusion of the kyphoscoliotic curve with posterior spinal instrumentation from T1 to L2. The Cobb angle postoperatively was 65° (Figure 3). The patient has neurologically fully recovered six months postoperatively.

## Discussion

In NF dysplasia causes severe wedging, strong rotation and scalloping of the vertebral bodies that result in a progressive abnormal spinal curvature [11]. Spindling of transverse processes, foraminal enlargement, saccular dilatations and pencilling of vertebral margins and apical ribs may contribute to costovertebral subluxation or dislocation of the rib head towards the neural foramen. A dumbbell intraspinal neurofibroma or dural ectasia may coexist. Spinal cord compression may be caused by vertebral angulation or tumorous lesions around the spinal canal [12,13].

The painful rib hump is an important clinical sign that is introduced for the first time by this report. A careful search for this sign is recommended. There is a high index of suspicion of an intraspinal rib displacement at the apex of a progressive short, sharp, angular, kyphoscoliotic curve when a region sensitive to pressure is clinically detected.

Pain is attributed to the mobile rib head and it should be considered as a warning sign for rib dislocation. The dislocated rib head when pressed against the thorax at the scoliotic apex during clinical examination irritates the adjacent nerve root into the enlarged neural foramina and produces pain. An acute violent displacement of the mobile rib head inside the spinal canal may traumatize the spinal cord which may result in neurological impairment. Patients with intraspinal rib displacement are at high risk for neurological injury after a fall with their back on the ground [10]. A strong hit on the rib hump may also lead in paraplegia.

CT-scan is a valuable diagnostic tool for the evaluation of the dystrophic osseous lesions in neurofibromatic spinal curves. Thin slices at the scoliotic apex are necessary for estimation of the anatomic relationship between the rib heads and the vertebral bodies. Specificity of MRI is very low in identifying the intraspinal rib displacement.

Although combined anterior and posterior spinal fusion is recommended [11,14] for dystrophic spinal curves, posterior fusion alone was sufficient for our patient and rib head was easily been resected through the posterior approach.

Spinal fusion in kyphoscoliosis due to NF should be performed early in order to prevent cord compression resulting in paraplegia. Rib displacement in NF scoliotic patients should be recognized early and resection of the head of an encroaching rib should be performed to minimize the neurological risk at the time of surgery for the spinal curvature correction and fusion.

## Authors' contributions

AG conceived the idea of the presented new clinical sign, was the clinical supervisor, performed the part of literature review and revised the manuscript for important intellectual content. SH, EV, MK and GK performed part of literature review and built the structure of the paper. EV also contributed by reviewing, text editing and adding certain parts of the manuscript. All authors have read and approved the final manuscript.
